# Host-plant induced changes in microbial community structure and midgut gene expression in an invasive polyphage (*Anoplophora glabripennis*)

**DOI:** 10.1038/s41598-018-27476-0

**Published:** 2018-06-25

**Authors:** Erin D. Scully, Scott M. Geib, Charles J. Mason, John E. Carlson, Ming Tien, Han-Yi Chen, Scott Harding, Chung-Jui Tsai, Kelli Hoover

**Affiliations:** 1 Stored Product Insect and Engineering Research Unit, USDA-ARS Center for Grain and Animal Health Research, Manhattan, KS 66502 USA; 2 Tropical Crop and Commodity Protection Research Unit, USDA-ARS Daniel K. Inouye Pacific Basin Agricultural Research Center, Hilo, HI 96720 USA; 3 Department of Entomology and Center for Chemical Ecology, The Pennsylvania State University, University Park, PA 16802 USA; 4 The Schatz Center for Tree Molecular Genetics, Department of Ecosystem Science and Management, The Pennsylvania State University, University Park, PA 16802 USA; 5 Department of Bioenergy Science and Technology (World Class University), Chonnam National University, Buk-Gu, Gwangju 500-757 Korea; 6 Department of Biochemistry and Molecular Biology, The Pennsylvania State University, University Park, PA 16802 USA; 7 Warnell School of Forestry and Natural Resources, University of Georgia, Athens, GA 30602-2152 USA; 8 Department of Genetics, University of Georgia, Athens, GA 30602-7223 USA; 9 Present Address: Plants for Human Health Institute, North Carolina State University, Kannapolis, NC 28081 USA

## Abstract

Polyphagous insect herbivores possess diverse mechanisms to overcome challenges of feeding in multiple plant species including, but not limited to, transcriptional plasticity and associations with obligate or facultative symbionts. The Asian longhorned beetle (*Anoplophora glabripennis*) is a polyphagous wood-feeder capable of developing on over 100 tree species and, like other polyphages, its genome contains amplifications of digestive and detoxification genes. This insect also possesses a diverse gut microbial community, which has the metabolic potential to augment digestive physiology. While the genomic repertoires of *A*. *glabripennis* and its microbial community have been studied previously, comparatively less is known about how the gut transcriptome and community change in response to feeding in different hosts. In this study, we show that feeding in two suitable hosts (*Acer* spp. and *Populus nigra*) altered the expression levels of multicopy genes linked to digestion and detoxification. However, feeding in a host with documented resistance (*Populus tomentosa*) induced changes in the transcriptome and community beyond what was observed in insects reared in *P*. *nigra*, including the downregulation of numerous β-glucosidases, odorant binding proteins, and juvenile hormone binding proteins, the upregulation of several cuticular genes, and the loss of one major bacterial family from the gut community.

## Introduction

Polyphagous insect herbivores must contend with the challenges of feeding on host plants with varying nutrient and allelochemical compositions. Behavioral, transcriptional, and physiological plasticity are integrated mechanisms used by polyphages to overcome these challenges. Evidence for this plasticity is apparent in the genomes and transcriptomes of many of these insects, which often contain large expansions of genes linked to digestion, detoxification, and stress responses whose expression can be modulated to respond to different plant hosts^1–3^. The peritrophic matrix (PM) also plays an important role in polyphagy and host range; its ability to provide a protective barrier from ingested defensive compounds is likely a major determinant in the outcome of plant-insect interactions^1^. Additionally, some plant defenses can target and disrupt the PM^4^, so its ability to remodel and repair itself is an important counter to plant defenses^5^. Insects also possess bacterial and fungal symbionts that can influence nutritional and defensive outcomes. For example, insect microbial symbionts can metabolize host plant defense chemicals, attenuate or suppress plant responses, and provision hosts with essential nutrients or digestive processes^6–13^. These microbial contributions may enable expansion of host ranges by allowing insects to feed on chemically defended hosts and/or hosts low in essential nutrients^14^.

The Asian longhorned beetle (*Anoplophora glabripennis*) is a wood-boring cerambycid native to China that has been introduced to North America and Europe. This insect is a broad generalist, documented to attack approximately 100 species comprising at least 40 different families throughout its geographic range^15^. Like other polyphages^1,2,16^ and xylophages^17,18^, amplifications of gene families coding for enzymes that facilitate digestion and detoxification are prominent throughout the *A*. *glabripennis* genome and gut transcriptome^3,19,20^. Carboxylesterases, UDP-glucuronosyltransferases  (UGTs), and glycoside hydrolase (GH) family 1 genes are particularly enriched in *A*. *glabripennis* relative to other insects^3,19^ and the expression patterns of these genes are plastic^21^. In concert with endogenous enzymes encoded by *A*. *glabripennis*, the gut microbial community also facilitates lignocellulose metabolism^22,23^ fixes and recycles nitrogen, and provisions essential amino acids^22–26^. Although the composition of the gut bacterial and fungal communities can vary between individuals at the operational taxonomic unit (OTU) level^26^, the taxonomic makeup and metabolic potential of the microbial community are similar across populations and a fungal member in the *Fusarium solani* species complex (FSSC) is consistently associated with this insect^22,27–30^. Some of these gut microbes appear to be transferred from mother to offspring^30,31^.

Although amplifications and transcriptional plasticity of genes linked to detoxification and digestion facilitate polyphagy, the majority of studies monitoring gene expression patterns in insects feeding in multiple hosts have focused on folivores^1,2^. Likewise, studies on the contributions and plasticity of microbial communities to polyphagy are similarly limited to folivores and piercing/sucking insects^10,11,32,33^. Overall, little is known about how feeding in different hosts impact gene expression or associated microbial communities in wood-feeding polyphages. This knowledge gap is notable given that *A*. *glabripennis* and some other wood-feeders have the potential to initiate biodeterioration in forest ecosystems^34^ by feeding on a broad range of both stressed and healthy hardwood trees^35^.

In this study, we assessed impacts on *A*. *glabripennis* larval gut gene expression and the gut microbial consortia in response to feeding in hosts belonging to two different genera, *Acer* and *Populus*. *Acer* spp. include many of *A*. *glabripennis’* most common hosts in the United States, while *Populus* spp. [Salicaceae] vary in their susceptibility to herbivore pressure, presumably in part due to the production of bitter, reactive phenolics^36–38^. We also evaluated the impacts of feeding in a host exhibiting field-level resistance on *A*. *glabripennis* and its gut community. For this experiment, larval *A*. *glabripennis* were reared in two *Populus* species: black poplar (*Populus nigra*) and Chinese white poplar (*Populus tomentosa*). *P*. *nigra* is commonly grown in the U.S. and China as a hybrid with other poplars for pulp, lumber, and as a biofuel source^39^ and has previously incurred damage in the field from *A*. *glabripennis*^40^. In contrast, Chinese white poplar (*P*. *tomentosa*) is considered resistant to this insect in China^37,41^. Thus, this study examined host plant induced changes to the *A*. *glabripennis* gut microbial community and transcriptome profiles in suitable host species belonging to two different genera and a host that is resistant in the field.

## Results

### Larvae Actively Fed on Both *P*. *nigra* and *P*. *tomentosa* After Insertion

Larvae actively fed on both poplar species as indicated by the appearance of frass. In addition, the guts of all recovered larvae used for RNA-Seq and amplicon analyses contained wood and ITS sequences derived from poplar, indicative of feeding. There were no major differences in survival rates between the insects reared in *P*. *nigra* and *P*. *tomentosa* and none of the insects molted during the experiment. Therefore, the insects collected were of approximately the same developmental stage both prior to insertion and after removal from the poplar trees. Additionally, no differences in relative growth rate were observed between the insects reared in *P*. *nigra* and *P*. *tomentosa*.

### RNA-Seq Analysis Revealed Divergent Impacts on Midgut Gene Expression of Insects Feeding in *P*. *nigra* and *P*. *tomentosa*

**Figure 1:**
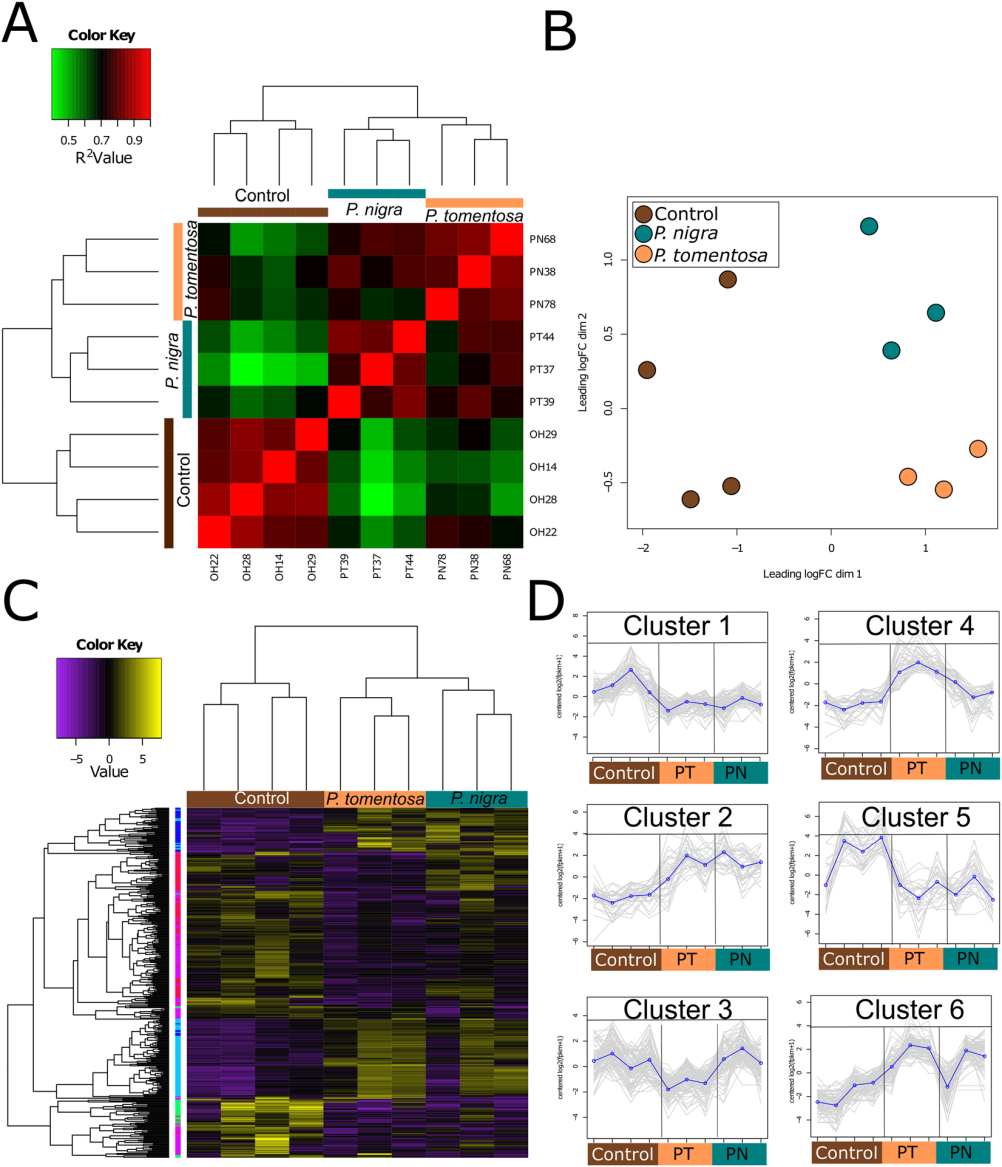
RNA-Seq Profiles of Guts of Third Instar *A*. *glabripennis* Feeding on *Acer spp*., *P*. *nigra*, or *P*. *tomentosa*. (**A**) Pearson’s Correlation Analysis of RNA-Seq Profiles from *A*. *glabripennis* Guts. Pearson’s correlation analysis of Euclidean dissimilarities of RNA-Seq profiles from beetles feeding in three substrates was performed using the ‘cor’ command in R^85^. Red represents high correlations (0.8 and above) while green represent low correlations (0.7 and below). n = 4 for *Acer* spp (control), n = 3 for *P*. *tomentosa*, and n = 3 for *P*. *nigra*. (**B**) NMDS Analysis of RNA-Seq Profiles from *A*. *glabripennis* Guts. NMDS analysis was conducted using the plotMDS.dge command in edgeR^86^. Prior to analysis, values were log2 transformed, z-score standardized, and genes with less than one count per million in at least three samples were removed from the count matrix. (**C**) Heatmap Analysis of Differentially Expressed Genes in Guts of *A*. *glabripennis*. Differentially expressed genes were identified using edgeR^86^ with an FDR ≤ 0.05 and a fold-change cutoff of ±2. Prior to heatmap analysis, values were log2 transformed and z-score standardized. Cluster analysis of samples and genes was performed based on Euclidean dissimilarities using the complete linkage method and the heatmap was prepared using the heatmap.3 package in R^85^. Purple indicates low expression, yellow indicates high expression, and sidebar indicates clusters of genes with similar expression profiles based on a k-means analysis. (**D**) Major Expression Profiles of Differentially Expressed Genes. A k-means analysis was used to identify six groups of genes that showed similar expression patterns across the three treatments. Cluster one represents genes downregulated in both poplar species (n = 110 genes) while cluster two represents genes upregulated in both poplar treatments (n = 40) compared to controls. Clusters three (n = 100) and four (n = 45) represent genes down- and up-regulated specifically in the *P*. *tomentosa* treatment, respectively. Cluster five (n = 30) contains genes upregulated in most of the controls, with the exception of one, while Cluster six (n = 85) contains genes that were more highly expressed in all individuals feeding in poplar with the exception of one individual from the *P*. *nigra* treatment.

RNA-Seq analysis revealed that feeding in both poplars had significant impacts on gene expression in the guts of *A*. *glabripennis* larvae. Correlation and non-metric multidimensional scaling (NMDS) analyses clearly separated the RNA-Seq profiles from the guts of larvae feeding on poplars from those feeding in the control logs along NMDS axis 1 (Fig. 1A and B). Importantly, the RNA-Seq profiles of the replicates belonging to each of the treatments were clearly separable from one another and the correlation values among the biological replicates within each treatment were high (R^2^ ≥ 0.80). These findings indicate that effects of collecting the insects from two different *Acer* spp. (control) on the RNA-Seq profiles were likely minimal and that major impacts on gene expression were associated with feeding in the poplar trees. Differential expression analysis using the Likelihood Ratio Test (LRT) in DESeq2 determined that the expression levels of 371 genes (False Discovery Rate (FDR) ≤ 0.05; fold-change ≥±2.0) were impacted by feeding in the two poplar species, of which, expression levels of 150 genes were specifically altered by feeding in *P*. *tomentosa*. In contrast, none were exclusively impacted by feeding in *P*. *nigra*.

Two-dimensional hierarchical clustering analysis using a k-means approach was used to identify six clusters containing genes with similar expression patterns across the three treatments (Fig. 1C and D). Cluster one contained approximately 110 genes whose expression levels were predominantly lower in guts of *A*. *glabripennis* feeding in the two poplars compared with the controls, while cluster two contained approximately 40 genes whose expression levels were elevated in the two poplar treatments in comparison to controls (Fig. 1D). Clusters three (100 genes) and four (45 genes) contained genes whose expression levels were exclusively lower and higher in the guts of individuals feeding in *P*. *tomentosa* compared to both *P*. *nigra* and the controls, respectively (Fig. 1D). Cluster five contained 30 genes whose expression was higher in the majority of the control samples compared to the individuals feeding in the two poplars, while cluster six contained 85 genes whose expression levels were elevated in all insects feeding in poplar with the exception of one individual from *P*. *nigra* (Fig. 1D and Supplementary Info).

### Expression Levels of Multicopy Genes with Hypothesized Involvement in Digestion and Detoxification were Impacted in the *P*. *nigra* and *P*. *tomentosa* Treatments

**Figure 2:**
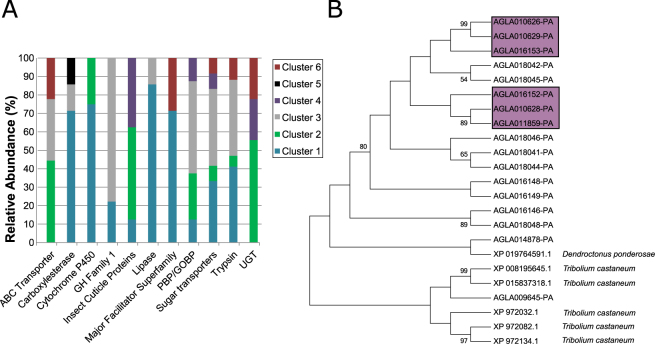
Relative Abundance of Genes in Various Functional Categories. (**A**) Relative Abundance of Gene Categories whose Expression Levels were Commonly Impacted by the Two Poplar Treatments. Genes were grouped into categories based on Pfam domain annotations. Pfams that were detected ≥6 times in the list of differentially expressed genes were defined as ‘common’. PBP/GOBP = Pheromone Binding Protein/General Odorant Binding Protein; UGT = UDP-glucuronsyltransferase; GH = Glycoside hydrolase. (**B**) GH  1 Genes Downregulated in Larvae Reared in *P*. *tomentosa*. Several GH  1 genes belonging to a large *A*. *glabripennis* specific expansion were downregulated exclusively in larvae feeding in *P*. *tomentosa* (highlighted in purple). These genes are distantly related to GH 1 genes found in the genomes of *Dendroctonus ponderosae* and *Tribolium castaneum* via neighbor-joining analysis and may have evolved in a lineage-specific manner.

Genes whose expression levels were most commonly impacted in this experiment included multi-copy genes, such as trypsins (17 genes), carboxylesterases (14 genes), GH  1 (11 genes), sugar (and other) transporters (11 genes), ABC transporters (nine genes), cytochrome P450s (eight genes), insect cuticle proteins (seven genes), lipases (seven genes), major facilitator superfamily proteins (seven genes), pheromone binding protein/general odorant binding protein family (PBP/GOBP; seven genes), and UDP-glucuronosyltransferases (UGTs; six genes) (Fig. 2A). Many of these genes were previously induced in insects feeding in *A*. *saccharum* (sugar maple) relative to a nutrient rich artificial diet, suggesting that they are important for digestion and plant interactions^21^ (Table 1 and Supplementary Info [Supplemental Table 1]).

**Table 1:** Most common types of genes whose expression levels were altered by feeding in poplar.

Cluster #	Gene ID	Annotation	Expression Altered in Sugar Maple versus Diet Experiment?	Direction of Expression Change (SM v Diet)
1	AGLA000186	Carboxylesterase	Y	Up
1	AGLA000622	Carboxylesterase	Y	Up
1	AGLA021994	Carboxylesterase	Y	Up
1	AGLA013181	Carboxylesterase	Y	Up
1	AGLA013187	Carboxylesterase	N	ND
1	AGLA000623	Carboxylesterase	Y	Up
1	AGLA006755	Carboxylesterase	Y	Up
1	AGLA013186	Carboxylesterase	Y	Up
1	AGLA001398	Carboxylesterase	Y	Up
1	AGLA000624	Carboxylesterase	Y	Up
1	AGLA007086	Cytochrome P450	Y	Down
1	AGLA001981	Cytochrome P450	Y	Up
1	AGLA008876	Cytochrome P450	N	ND
1	AGLA001983	Cytochrome P450	Y	Up
1	AGLA008875	Cytochrome P450	N	ND
1	AGLA008874	Cytochrome P450	N	ND
1	AGLA009630	Glycosyl Hydrolase Family 1	Y	Up
1	AGLA016148	Glycosyl Hydrolase Family 1	Y	Up
1	AGLA020447	Insect Cuticle Protein	NE	NE
1	AGLA011978	Lipase	Y	Up
1	AGLA011979	Lipase	N	ND
1	AGLA010459	Lipase	N	ND
1	AGLA011980	Lipase	N	ND
1	AGLA011977	Lipase	Y	Up
1	AGLA010457	Lipase	Y	Down
1	AGLA005008	Major Facilitator Superfamily	N	ND
1	AGLA005007	Major Facilitator Superfamily	Y	Up
1	AGLA005009	Major Facilitator Superfamily	N	ND
1	AGLA000050	Major Facilitator Superfamily	Y	Down
1	AGLA005004	Major Facilitator Superfamily	Y	Up
1	AGLA009811	Pheromone Binding Protein/General Odorant Binding Protein (PBP/GOBP)	NE	NE
1	AGLA014591	Sugar (and other) Transporter	Y	Down
1	AGLA003928	Sugar (and other) Transporter	N	ND
1	AGLA017528	Sugar (and other) Transporter	N	ND
1	AGLA008621	Sugar (and other) Transporter	N	ND
1	AGLA008645	Trypsin	Y	Up
1	AGLA001399	Trypsin	Y	Up
1	AGLA014946	Trypsin	Y	Up
1	AGLA004632	Trypsin	Y	Up
1	AGLA005309	Trypsin	Y	Up
1	AGLA017002	Trypsin	Y	Up
1	AGLA017633	Trypsin	N	Up
1	AGLA002744	Trypsin	N	Up
2	AGLA014208	ABC Transporter	Y	Down
2	AGLA012319	ABC Transporter	N	ND
2	AGLA017554	ABC Transporter	N	ND
2	AGLA006710	ABC Transporter	N	ND
2	AGLA020767	Cytochrome P450	N	ND
2	AGLA007077	Cytochrome P450	N	NE
2	AGLA009638	Glycosyl Hydrolase Family 1	Y	Up
2	AGLA018084	Glycosyl Hydrolase Family 1	N	ND
2	AGLA013838	Insect Cuticle Protein	N	ND
2	AGLA005871	Insect Cuticle Protein	N	ND
2	AGLA017561	Insect Cuticle Protein	N	ND
2	AGLA020564	Insect Cuticle Protein	N	ND
2	AGLA006507	PBP/GOBP	Y	Up
2	AGLA009822	PBP/GOBP	N	ND
2	AGLA003177	Sugar (and other) Transporter	N	ND
2	AGLA005865	Trypsin	N	ND
2	AGLA003185	UDP-Glucuronsyltransferase	Y	Down
2	AGLA006593	UDP-Glucuronsyltransferase	N	ND
2	AGLA003186	UDP-Glucuronsyltransferase	Y	Down
2	AGLA010474	UDP-Glucuronsyltransferase	N	NE
2	AGLA017105	UDP-Glucuronsyltransferase	N	NE
3	AGLA010510	ABC Transporter	N	ND
3	AGLA015335	ABC Transporter	N	ND
3	AGLA010186	ABC Transporter	N	ND
3	AGLA000625	Carboxylesterase	Y	Up
3	AGLA013190	Carboxylesterase	Y	Up
3	AGLA010628	Glycosyl Hydrolase Family 1	Y	Up
3	AGLA011859	Glycosyl Hydrolase Family 1	Y	Up
3	AGLA016153	Glycosyl Hydrolase Family 1	Y	Up
3	AGLA010626	Glycosyl Hydrolase Family 1	Y	Up
3	AGLA016152	Glycosyl Hydrolase Family 1	Y	Up
3	AGLA011858	Glycosyl Hydrolase Family 1	N	ND
3	AGLA010629	Glycosyl Hydrolase Family 1	Y	Up
3	AGLA002308	Lipase	Y	Up
3	AGLA009821	PBP/GOBP	Y	Up
3	AGLA009820	PBP/GOBP	Y	Up
3	AGLA009817	PBP/GOBP	N	NE
3	AGLA009823	PBP/GOBP	Y	Up
3	AGLA003176	Sugar (and other) Transporter	N	ND
3	AGLA012578	Sugar (and other) Transporter	N	ND
3	AGLA000228	Sugar (and other) Transporter	N	ND
3	AGLA000052	Sugar (and other) Transporter	N	ND
3	AGLA006155	Sugar (and other) Transporter	N	ND
3	AGLA000992	Trypsin	Y	Up
3	AGLA010158	Trypsin	Y	Up
3	AGLA008741	Trypsin	Y	Up
3	AGLA017632	Trypsin	N	ND
3	AGLA014440	Trypsin	N	ND
3	AGLA006989	Trypsin	N	ND
3	AGLA001394	Trypsin	Y	Up
3	AGLA001741	Trypsin	Y	Up
4	AGLA020448	Insect Cuticle Protein	N	NE
4	AGLA020447	Insect Cuticle Protein	N	NE
4	AGLA016071	Insect Cuticle Protein	N	ND
4	AGLA009811	PBP/GOBP	N	NE
4	AGLA019820	Sugar (and other) Transporter	N	NE
4	AGLA004754	UDP-Glucuronsyltransferase	N	NE
4	AGLA017105	UDP-Glucuronsyltransferase	N	NE

Previously, several categories of genes were hypothesized to aid in digestion, detoxification, nutrient acquisition, and host range determination in *A*. *glabripennis* and expression levels of these genes in the gut were altered as larvae fed in *Acer saccharum*^21^ compared to a nutrient rich artificial diet high in nutrients and free of plant defensive compounds. Expression levels of several of these genes were also altered as larvae fed on poplar. Shown are the cluster assignments and whether or not the expression of these genes was altered in the previous sugar maple versus diet feeding experiment for gene families that appeared ≥6 times in the list of 371 differentially expressed genes. PBP/GOBP = Pheromone binding protein/general odorant binding protein; Up = upregulated in insects reared in *A*. *saccharum*; Down = down-regulated in insects reared in *A*. *saccharum*. ND = no difference; NE = not expressed in sugar maple or artificial diet reared insects. Cluster 1 = downregulated in both poplar treatments; 2 = upregulated in both poplar treatments; 3 = downregulated in the *P*. *tomentosa* treatment; 4 = upregulated in the *P*. *tomentosa* treatment.

### Genes Coding for Polygalacturonases and Other Digestive Genes were Downregulated in Larvae Feeding in Both Poplars (Cluster 1)

A single GO term, polygalacturonase activity (GO:0004650) (Table 2), was enriched in genes downregulated in larvae feeding in both poplars (cluster 1), which included three GH 28 genes with *in vitro* polygalacturonase activity^3^. Cluster 1 also included the largest number of carboxylesterases (10 genes, including seven that belong to an *A*. *glabripennis*-specific expansion)^3^, trypsins (seven genes), lipases (six genes) and cytochrome P450s (six genes). Additional genes coding for digestive enzymes that were assigned to this group included one GH 45 gene capable of functioning as an endo-β-1,4-glucanase *in vitro*^3,20^, two GH 30 genes previously upregulated in larvae feeding in sugar maple trees compared to artificial diet^21^, one cysteine proteinase, and one GMC oxidoreductase. Likewise, genes coding for detoxification enzymes and other genes coding for enzymes involved in stress response were assigned to this group, including one gene annotated as aldehyde oxidase/xanthine dehydrogenase, a glutathione peroxidase, a glutathione S-transferase (GST), one peptide methionine sulfoxide, which can repair proteins inactivated by oxidation, two HSP20 genes, which can protect proteins against stress-induced proteolysis and denaturation, one BAG domain protein, and one E3 ubiquitin protein ligase. The same glutathione peroxidase, GST, peptide methionine sulfoxide, HSP20 genes, and BAG domain gene were upregulated previously in the guts of insects feeding in sugar maple compared to those feeding in artificial diet^21^.

**Table 2:** Gene ontology (GO) enrichment analysis.

Cluster	P-value	Total Number of Differentially Expressed Genes	Total Number of Genes in Category	Term
1	0.037	3	3	GO:0004650 Polygalacturonase
4	0.0046	4	6	GO:0042302: Structural Constituent of Cuticle
5	0.036	1	1	GO:00019787: Ubiquitin-like protein transferee activity
5	0.039	2	8	GO:0005974: Carbohydrate Metabolic Process
6	0.024	2	2	GO:0003774: Motor Activity
6	0.0272	4	6	GO:0016773: Phosphotransferase Activity
6	0.044	3	4	GO:0006811: Ion Transport

GO enrichment analysis was performed using GoSeq to identify enriched terms in genes that shared similar expression patterns across the three treatments (Clusters 1–6). In all cases, the list of genes with detectable expression levels in at least two samples across all three treatments was used as a reference to determine enrichment. Cluster 1 = downregulated in both poplar treatments; 4 = upregulated in the *P*. *tomentosa* treatment; 5 = upregulated in all but one of the controls; 6 = upregulated in all individuals feeding in poplar with the exception of one individual from the *P*. *nigra* treatment. No enriched categories were detected in clusters 2 (upregulated in both poplar treatments) or 3 (downregulated in the *P*. *tomentosa* treatment).

### Genes Coding for UDP-Glucuronosyltransferases (UGTs) and Structural Proteins were Upregulated in Both Poplar Treatments (Cluster 2)

In contrast to the genes downregulated in both poplar treatments, genes upregulated in both poplar treatments (cluster 2) included only a single gene with predicted digestive roles. One GH 18 chitinase was highly expressed in insects reared in both poplars; however, it was not previously induced in the sugar maple and artificial diet comparison and, instead, may have roles in PM remodeling^21^. Supporting impacts to genes coding for structural components of the gut tissue, this group of genes was enriched with a single GO term, structural constituent of cuticle (GO:0043202) (Table 2), which included four cuticular proteins. Importantly, various regions of the guts of phytophagous beetles are often lined with a relatively impermeable cuticular layer to protect endothelial cells from physical or chemical damage^42^. Interestingly, the largest numbers of UGTs (five genes, three of which were found in a tandem array on the same genomic scaffold) were also assigned to this group of genes, while other genes with predicted roles in stress response and detoxification were also upregulated in both poplar treatments. These genes included one aldehyde dehydrogenase, which can serve detoxification roles, one poly (adp-ribose) polymerase involved in DNA repair and programmed cell death, and two *ham1* family genes, which can protect cells from mutagenic base analogs that can be formed spontaneously *in vivo* from CYP450 activity and/or exposure to UV or radical oxygen species.

### Genes Coding for GH Family 1 and General Odorant Binding Proteins were Predominantly Downregulated in the *P*. *tomentosa* Treatment (Cluster 3)

The highest numbers of GH family 1 genes (seven genes) were downregulated exclusively in larvae feeding in *P*. *tomentosa*. Notably, all of these GH 1 genes were part of an *A*. *glabripennis* specific expansion (Fig. 2B) relative to other beetle genomes. Other digestive genes that were downregulated included sugar (and other) transporters (five genes), seven trypsin genes, a GMC oxidoreductase, and a single GH 38 gene annotated as α-mannosidase. The largest number of PBP/GOBPs (five genes) were also downregulated exclusively in larvae feeding in *P*. *tomentosa*. Although volatiles are unlikely to be found in guts, PBP/GOPs are often expressed in non-olfactory tissues and are thought to bind and transport hydrophobic compounds, including juvenile hormone, or help regulate immune pathways^43,44^. Two genes coding for GH family 18 chitinases and one gene coding for a GH 16 beta-1,3-glucan binding protein were downregulated in the *P*. *tomentosa* treatment. In contrast to the GH 18 chitinase whose expression was upregulated in insects reared in poplar, the two GH 18 genes in this cluster were previously induced in sugar maple fed larvae. Because they lacked carbohydrate binding domains required for interaction with the PM, these enzymes were hypothesized to be important for digestion of fungal chitin or chitin from other dietary sources^21^. The GH 16 protein assigned to this expression module contains a carbohydrate binding domain (CBM family 32) and is likely important for the detection and recognition of microbes^45^.

Additionally, a single gene annotated as ecdysteroid kinase was also downregulated in the *P*. *tomentosa* treatment and no genes annotated as ecdysteroid kinases were detected in any other expression profiles. Other genes downregulated in the *P*. *tomentosa* treatment linked to developmental processes included a gene annotated as farnesol dehydrogenase, which is involved in synthesizing juvenile hormone. Several genes involved in stress responses were downregulated including the antioxidant enzyme peroxiredoxin, E3 ubiquitin ligase, and lysosomal thiol reductase.

### Genes Coding for Structural Proteins and Enzymes Linked to Fatty Acid Metabolism were Upregulated in Larvae fed in *P*. *tomentosa* (Cluster 4)

Three genes coding for cuticular proteins along with two genes homologous to *yellow* in Drosophila and one gene homologous to *peritrophin A* were upregulated in insects feeding in *P*. *tomentosa* (cluster 4) compared to the other two feeding treatments. Previously, yellow proteins have been linked to production of black melanin in *Drosophila*^46^. The production of black melanin has been linked to cuticle hardening and stress response; thus, this protein may have similar roles in *A*. *glabripennis*^47^. Further, two UGTs, one HSP70, one aldo-keto reductase (AKR), one ubiquitin conjugating enzyme for proteolysis, and one alcohol dehydrogenase with likely roles in detoxification and/or elimination of damaged proteins from the gut were specifically upregulated in individuals feeding in *P*. *tomentosa*. Finally, one PBP/GOBP, and one juvenile hormone binding protein (JHBP) were upregulated exclusively in these individuals. Annotations for genes associated with clusters five and six are presented in the Supplementary Info.

### Feeding in *P*. *tomentosa* and *P*. *nigra* Impacted Bacterial Community Structure

**Figure 3:**
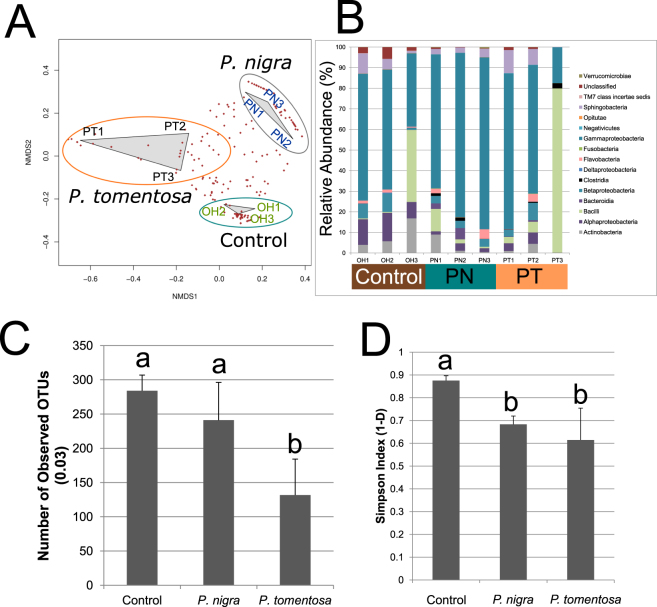
Impacts of Feeding in Different Poplar Species on 16S Bacterial Communities. (**A**) Non-metric Multidimensional Scaling (NMDS) Analysis of 16S Gut Bacterial Communities. NMDS analysis was performed on subsampled data using the metaMDS command from the vegan library (R)^85^. Brown dots represent the OTUs and their proximity to each treatment indicates their association with each sample; OH = control, PN = *P*. *nigra*, PT = *P*. *tomentosa*. (**B**) Relative Abundances of Bacterial Taxonomic Classes. The relative abundances of each taxonomic class were computed by dividing the number of reads assigned to each class by the number of total number of reads obtained from each library. Ribosomal Database Project taxonomies were computed using the classify.otu command in mothur^78^. A confidence threshold of 80 was required for all taxonomic assignments. (**C**) Richness of 16S Gut Bacterial Communities in *A*. *glabripennis* Feeding in Controls, *P*. *nigra*, and *P*. *tomentosa*. 16S amplicon analysis was performed on the guts of individual *A*. *glabripennis* larvae feeding in maple logs collected from an infestation site in Bethel, OH (control; n = 3) and larvae inserted into *P*. *tomentosa* (n = 3) and *P*. *nigra* (n = 3). Richness was determined by counting the number of OTUs detected in each group after each sample was normalized by subsampling the same number of reads from each library. Statistical analysis was performed using ANOVA followed by Tukey’s HSD in R^85^ to test for pairwise differences. (**D**) Simpson Index of 16S Gut Bacterial Communities in *A*. *glabripennis* feeding in controls, *P*. *nigra*, and *P*. *tomentosa*. Simpson indices were computed using the ‘summary single’ command in mothur (1.34.1) on subsampled data (as described above) and statistical analysis was performed using ANOVA followed by Tukey’s HSD in R^85^.

NMDS analysis indicated that the three biological replicates within each treatment clustered together and that the gut communities associated with the three feeding treatments were clearly separable from one another (Fig. 3A). Further, hierarchical clustering analysis indicated that the three biological replicates from each treatment were correlated with one another (Supplementary Info [Supplementary Fig. 1]). The differences in community structure were supported by AMOVA and HOMOVA analyses (F_2,6_ = 3.52, p = 0.014) with *P*. *tomentosa* versus *P*. *nigra* being the only post-hoc pairwise comparison that differed significantly (F_1,4_ = 4.44, p < 0.001 and BValue = 0.428, p < 0.001), suggesting that feeding in the different poplar species impacted the structures of the microbial communities to different extents. In addition, the AMOVA p-value comparing the *P*. *nigra* and control treatments was marginally insignificant (F_1,4_ = 6.19, p = 0.07).

### Feeding in *P*. *tomentosa* and *P*. *nigra* Reduced Bacterial Community Diversity and had Minor Impacts on Taxonomic Composition

Overall, the community richness, taxonomic composition, and variation of the gut communities sampled from the control larvae strongly resembled those of communities from *A*. *glabripennis* sampled previously from the Penn State University colony and from several field sites, which were dominated by Gammaproteobacteria^22,23,26,48^. Despite the impacts of feeding in *P*. *tomentosa* on community structure, major disruptions to the taxonomic distribution of the gut communities at the class level were not observed (Fig. 3B). In most cases, the communities of all insects were dominated by Gammaproteobacteria, with the exception of one of the communities associated with the *P*. *tomentosa* treatment, which was dominated by Bacilli. The relative abundances of several other prominent classes also did not differ between feeding treatments, including Sphingobacteria and Bacilli, whose abundances were variable within all three treatments. However, lower relative abundances of several less prominent classes previously associated with the *A*. *glabripennis* gut community were noted in individuals reared in *P*. *tomentosa* and *P*. *nigra*, including Actinobacteria (3.5 ± 2.7% in *P*. *nigra*; 1.9 ± 1.3% in *P*. *tomentosa*; 8.5 ± 3.5% in controls) and Alphaproteobacteria (2.3 ± 0.6% in *P*. *nigra*; 3.1 ± 0.6% in *P*. *tomentosa*; 11.4 ± 1.8% in controls). Minor differences in taxonomic composition were also noted at finer taxonomic resolutions. For example, the relative abundance of families Xanthomonadaceae and Nocardioidaceae were significantly reduced in both poplar communities compared to the controls while members of the family Comamonadaceae were not detectable in the communities associated with the *P*. *tomentosa.* No other differences in taxonomic composition at any other rank (family, genus, etc.) were noted.

Although only minor impacts on taxonomic composition were observed, feeding in both poplars had major impacts on diversity and richness relative to the controls. Not only was the taxonomic richness reduced by approximately 50% from 250 ± 17 OTUs to 118 ± 41 OTUs in insects reared in *P*. *tomentosa* compared to the control treatment (Fig. 3C), but the Simpson and Shannon indices were significantly lower in individuals feeding in both *P*. *nigra* and *P*. *tomentosa* compared to the controls (Fig. 3D), suggesting that diversity and evenness had been impacted in insects feeding in both poplars. Other indices also supported reduced community diversities in the individuals feeding in poplar (Table 3). In addition, the richness of the majority of the bacterial classes identified in the gut was significantly lower in both poplars compared to the control. The only exception was Gammaproteobacteria, whose richness was unchanged in the two poplar treatments compared to the controls.

**Table 3:** Community Metrics for 16S and ITS Amplicons.

		Richness	Chao	Ace	Shannon	Simpson 1-D
16S	Control	135 ± 11(a)	260 ± 41(a)	330 ± 52(a)	3.13 ± 0.18(a)	0.87 ± 0.021(a)
	*P*. *nigra*	102 ± 22(ab)	184 ± 27(b)	281 ± 96(a)	2.22 ± 0.22(b)	0.69 ± 0.032(b)
	*P*. *tomentosa*	62 ± 23(b)	91 ± 45(c)	130 ± 96(b)	1.88 ± 0.54(b)	0.62 ± 0.15(b)
ITS	Control	44 ± 7(a)	46 ± 7(a)	49 ± 7(a)	2.04 ± 0.32(a)	0.80 ± 0.048(a)
	*P*. *nigra*	20 ± 6(b)	24 ± 8(b)	26 ± 9(b)	1.38 ± 0.24(b)	0.67 ± 0.074(b)
	*P*. *tomentosa*	23 ± 7(b)	25 ± 3(b)	27 ± 1(b)	1.80 ± 0.05(a)	0.76 ± 0.012(ab)

Mothur (v 1.34.3) was used to compute community metrics on subsampled reads. Values represent means and standard errors while values represented by different letters represent statistical differences within each indicator at p ≤ 0.05 using ANOVA followed by a Tukey HSD post hoc test. Analyses were performed separately for ITS and 16S amplicons.

Despite the variation in microbial community structure between individual beetles, 35 OTUs were consistently found in the control insects. To determine if any of these shared OTUs were specifically reduced in abundance and/or absent in the *P*. *tomentosa* treatment relative to the other two treatments (*P*. *nigra* and control), the random forests algorithm was utilized. The major OTUs absent from individuals feeding in both poplar species included OTU0004 (*Erwinia*), OTU0017 (unclassified Xanthomonadaceae), OTU0050 (unclassified Proteobacteria), OTU0053 (unclassified Bacteroidetes), OTU0055 (*Microbacterium*), OTU0072 (*Ochrobactrum*), OTU0076 (unclassified Microbacteriaceae), and OTU0089 (unclassified Proteobacteria). OTUs specifically absent in the *P*. *nigra* treatment relative to controls included OTU0002 (unclassified Enterobacteriaceae) and OTU0063 (*Acinetobacter*), while those absent specifically in the *P*. *tomentosa* treatment included OTU0048 (*Gemmobacter*) and OTU0078 (unclassified Sphingomonadaceae). Overall, these OTUs were the major drivers of the clustering patterns observed in Fig. 3B and no other consistent patterns regarding OTU loss or reduction in abundance were noted among the poplar treatments.

### Feeding in *P*. *tomentosa* and *P*. *nigra* Impacted Fungal Community Structure and Taxonomic Composition

**Figure 4:**
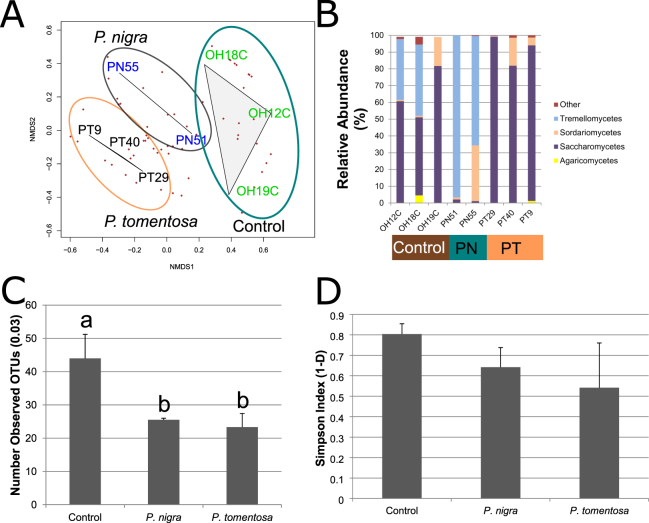
Impacts of Feeding in Different Poplar Species on ITS Fungal Communities. (**A**) Non-metric Multidimensional Scaling (NMDS) Analysis of ITS Gut Fungal Communities. NMDS analysis was performed on subsampled data using the ‘metaMDS’ command from the vegan library (R)^85^. Brown dots represent the OTUs and their proximity to each treatment indicates their association with each sample; OH = control, PN = *P*. *nigra*, PT = *P*. *tomentosa*. (**B**) Relative Abundances of Fungal Taxonomic Classes. The relative abundances of each taxonomic class were computed by dividing the number of reads assigned to each class by the number of total number of reads obtained from each library. Taxonomic classifications were obtained using the UNITE database and the ‘classify.otu’ command in mothur^78^. A confidence threshold of 90 was required for taxonomic assignments. ITS amplicon yields for the third *P*. *nigra* replicate were very low, so only two replicates are displayed. (**C**) Richness of ITS Gut Fungal Communities in *A*. *glabripennis* Feeding in Controls, *P*. *nigra*, and *P*. *tomentosa*. ITS amplicon analysis was performed on the guts of individual *A*. *glabripennis* larvae feeding in control logs collected from an infestation site in Bethel, OH (control; n = 3) and larvae inserted into *P*. *tomentosa* (n = 3) and *P*. *nigra* (n = 2). Richness was determined by counting the number of OTUs detected in each group after each sample was normalized by subsampling the same number of reads from each library. Statistical analysis was performed using ANOVA followed by Tukey’s HSD in R^85^ to test for pairwise differences. (**D**) Simpson Index of ITS Gut Fungal Communities in *A*. *glabripennis* feeding in controls, *P*. *nigra*, and *P*. *tomentosa*. Simpson indices were computed using the ‘summary.single’ command in mothur (1.34.1)^78^ on subsampled data (as described above) and statistical analysis was performed using ANOVA followed by Tukey’s HSD in R^85^. No significant differences were found.

To determine the impacts of the three feeding treatments on fungal gut communities, ITS amplicon analysis was performed on three insects from each treatment. Although fungal PCR products were identified in the majority of the amplicon libraries, one of the amplicon libraries from the *P*. *nigra* treatment did not yield significant numbers of fungal amplicons and was removed from the analysis. Consistent with previous studies^22,48^, NMDS analysis of the fungal communities revealed more variation among the community structures of the fungal communities in comparison to the bacterial communities, which is depicted by the larger distances between biological replicates from the same feeding treatment. However, the fungal community associated with the *P*. *tomentosa* treatment displayed considerably less variation in comparison (Fig. 4A). Despite these high within-treatment variations, the biological replicates from each of the three treatments were still more strongly correlated with one another than they were with samples from other treatments, suggesting that the major impacts to fungal communities were caused by our experimental treatments. While significant differences in community structure were detected via AMOVA (F_2,6_ = 1.44; p = 0.05), the post-hoc comparisons were marginally insignificant (p > = 0.10). No significant differences between the communities were detected using either ANOSIM or HOMOVA.

However, strong shifts in the taxonomic composition of the communities associated with the two poplar treatments were noted in comparison to the controls (Fig. 4B). For example, communities from the control insects were dominated by members of the classes Tremellomycetes and Saccharomycetes, which differed from fungal community compositions observed in previous studies where communities were typically dominated by Saccharomycetes^22,48^. In individuals feeding in *P*. *nigra*, the relative abundance of Saccharomycetes declined in comparison to the controls while the relative abundance of Tremellomycetes increased in these individuals. In contrast, members of Tremellomycetes were not detectable in any insects feeding in *P*. *tomentosa*, while the relative abundance of Saccharomycetes increased.

Fungal community richness in the control insects was similar to richness observed in previous studies^22,48^. Feeding in both poplars drastically reduced the fungal community richness by approximately 50% relative to the control treatment from 44 ± 8 OTUs to 26 ± 0.5 and 23 + 4 OTUs in *P*. *nigra* and *P*. *tomentosa*, respectively (Fig. 4C). In contrast to the 16S gut communities, diversity metrics were more variable for the fungal communities associated with the two poplar treatments. For this reason, there were no major differences between Shannon, Simpson (Fig. 4D), or other major ecological diversity indices among the insects associated with the three feeding treatments (Table 3). As in previous studies^22,48^, the relatively simple structure of the fungal community led to the detection of only ten OTUs that were shared among all of the control insects. Several of these OTUs were responsible for driving the clustering patterns observed in the NMDS plot. Specifically, OTU0001 (*Candida* spp.) was absent in both poplar treatments, OTU0002 (Tremellomycetes) was absent in individuals fed in *P*. *tomentosa*, and OTUs 0011 and 0012 (Saccharomycetales) were absent in individual reared in *P*. *nigra*. Furthermore, feeding in either poplar species did not significantly influence the relative abundance of Sordariomycetes or the OTU classified as FSSC (OTU006), which was present in approximately the same relative abundance in individuals from all three treatments. No other consistent patterns of OTU loss were observed in the two poplar treatments.

## Discussion

Polyphagous insects rely on transcriptional plasticity and, in some cases, facultative and/or obligate symbionts to overcome the challenges of feeding in multiple hosts. These mechanisms contribute important roles in *A*. *glabripennis’* ability to attack over 100 different broadleaf tree species with varying defense chemistries^15^. However, trees belonging to the same genus are not always uniformly susceptible to *A*. *glabripennis*, with some species being less vulnerable to infestation than others^40^. For example, some *Populus* spp. are considered susceptible to *A*. *glabripennis*, with field-level resistance documented in *P*. *tomentosa*^37,41^. In most cases, the mechanisms of resistance and their impacts on insect physiology are not known, but could be due in part to differences in nutrient composition or accessibility, differences in cell wall digestibility, the production of plant volatiles, the presence of digestive enzyme inhibitors, or the presence and/or differential abundance of constitutive or induced defense compounds^49^. In this study, we investigated how feeding in two susceptible hosts and one resistant host tree impacted gut gene expression and the gut microbial community of *A*. *glabripennis*. Overall, we documented differences in gut gene expression and microbial community composition that were associated with feeding in both *Populus spp*. as well as differences exclusively associated with feeding in *P*. *tomentosa*.

Unlike *Acer* spp., *Populus* spp. are capable of producing a diversity of phenolic-based plant defensive compounds that can have negative impacts on herbivore feeding and fitness. Among the most prominent of these compounds are the bitter salicinoids that are present in all poplar tissues. Salicinoids possess antifeedant properties and are also highly reactive, causing physical damage to tissues, proteins, and digestive enzymes^50–52^. Cell wall composition also varies between *Ace*r spp. and *Populus* spp. Specifically, the ratios of celluloses and hemicelluloses to lignin differ^53^ and pectin levels may also differ or may be methylated to different extents^54^.

These differences in defense compound profiles and cell wall composition could explain why the expression levels of several detoxification and digestive genes were commonly differentially expressed in insects feeding in both poplars (clusters 1 and 2) relative to the insects reared in *Acer* spp. For example, several PM-related and cuticular genes were upregulated in both poplar treatments, indicating that feeding in poplar may impact the structure of the PM or the cuticle, which serve to protect the insect and its tissues from mechanical damage or damage from ingested toxins^55^. Supporting this hypothesis, the expression levels of several genes coding for cuticular and PM related proteins were impacted in *Polygonia c-album* in response to plant hosts with differing defense compound profiles^1^ and the PM was found to be a target of maize toxins in *Spodoptera frugiperda*^56^. Additionally, UGTs were upregulated in the two poplar treatments, whose gene products have been linked to conjugative detoxification of plant defensive compounds in several other insect species^57^.

Surprisingly, however, many other genes coding for enzymes linked to detoxification and digestion were downregulated in both poplar treatments, including CYP450s, lipases, carboxylesterases, and glycoside hydrolases, and the expression of genes from primary metabolic pathways were not strongly impacted in either treatment. Strong transcriptional responses of genes linked to detoxification coupled with low transcriptional impacts to genes linked to primary metabolism have been observed previously in insects feeding on plant hosts with varying levels of toxicity^1,2,58^. Constitutive expression of genes from primary metabolic pathways coupled with dynamic expression of detoxification genes could be beneficial because it may allow insects to quickly adapt to environments where nutrient and defense compound compositions could change drastically^59^. Furthermore, the downregulation of detoxification genes can be adaptive under certain circumstances. For example, carboxylesterases can hydrolyze ester linkages in a broad range of substrates^60^ and ester hydrolysis of salicinoids was recently demonstrated as a first step in the metabolism and activation of these compounds in gypsy moth^52^. While alkaline conditions found in the guts of several insect species and in several regions of the *A*. *glabripennis* gut likely lead to the spontaneous hydrolysis of salicinoids and other phenolic defensive compounds^52^, carboxylesterases may also act on these compounds^61^. Therefore, lower expression of carboxylesterases could reduce activation of phenolic defensive compounds found in both *P*. *nigra* and *P*. *tomentosa*, particularly in the more acidic regions of the *A*. *glabripennis* anterior midgut where these compounds are likely more stable^48^. Lipases can also have esterase activity, but can also have roles in lipid metabolism/digestion, response to oxidative stress^60^, and mediating host plant defense responses^62^ and thus could be linked to differences in defense compound or nutrient profiles between *Acer* spp. and *Populus* spp. Genes annotated as polygalacturonases, trypsins, GH 45 cellulases, and two GH 30 genes whose functions are not known^3^ were also downregulated, which are likely attributable to differences in cell wall composition between *Acer* spp. and *Populus* spp.

The expression levels of several genes were impacted specifically in individuals feeding in *P*. *tomentosa* relative to insects feeding in both the control and *P*. *nigra* treatments. For example, large numbers of GH 1 and odorant binding protein (OBPs) genes were downregulated exclusively in the *P*. *tomentosa* treatment. Notably, these GH 1 genes are part of a large expansion of GH 1 genes in the *A*. *glabripennis* genome^3^ relative to *Tribolium castaneum* and *Dendroctonus ponderosae*. Although the precise function of these genes is not known in *A*. *glabripennis* or in other insects, many GH 1 enzymes function as β-glucosidases, which can hydrolyze and activate salicinoids and other phenolic defensive compounds^63^. OBPs are predominantly known for their roles in odor and taste perception; however, they are often expressed in non-olfactory tissues where they have been shown to mediate interactions with symbionts and are also hypothesized to bind and transport small hydrophilic compounds, such as hemocyanins and small chain fatty aphids. Additional roles could include modulation of phenol oxidase or other defense pathways and the regulation of digestive processes. Further, several cuticular and PM-related genes were also upregulated specifically in insects reared in *P*. *tomentosa*. The differential expression of these genes in *A*. *glabripennis* larvae could be a response to variations in defensive compound profiles produced by *P*. *tomentosa* relative to *P*. *nigra*.

Feeding in *P*. *nigra* and *P*. *tomentosa* predominantly impacted the gut bacterial communities of *A*. *glabripennis* larvae. Reductions in the richness, diversity, and evenness of the bacterial communities were observed in individuals fed in *P*. *tomentosa* while the structure of the bacterial community differed among all three treatments. Despite the fact that only minor disruptions to the taxonomic composition were observed, the abundances of several taxa with predicted roles in digestion and nutrient acquisition were altered in the poplar treatments. For example, members of the family Comamonadaceae, which have been consistently detected in other *A*. *glabripennis* populations and often express genes linked to the synthesis of essential aromatic and branched chain amino acids in the gut^22,23^, were completely absent in individuals feeding in *P*. *tomentosa*. Additionally, members of the bacterial family Xanthomonadaceae were absent in both poplar treatments relative to the controls. In previous studies, members of the family Xanthomonadaceae were transcriptionally active in the *A*. *glabripennis* gut and expressed genes coding for enzymes linked to 5-carbon sugar metabolism (ie, xylose) and cell wall digestion (Xanthomonadaceae)^22,23,26^ Although these taxonomic groups can make important contributions to digestive physiology, the exact consequences that their absences have on larval fitness are not known. Many members of the gut community have potential functional redundancies^21,22^ and thus, it is possible that metabolic pathways encoded by OTUs that were unaffected by the feeding treatments could compensate for the physiological functions of OTUs that were lost. Further, feeding in different hosts could have impacts on the transcriptional activities of other OTUs whose abundances were not impacted; however, RNA yields were insufficient to measure these responses.

Fungal richness was also reduced in both poplar treatments and the taxonomic composition was altered. The most major impacts were on the abundances of environmental fungi belonging to the Tremellomycetes, which were absent in the *P*. *tomentosa* treatment, but more abundant in the *P*. *nigra* treatment. Members of the class Tremellomycetes have not been previously detected in *A*. *glabripennis* guts and are predominantly environmental saprophytic fungi that likely colonized control logs in the field. Thus, they likely represent transients in this system. Although all individuals inserted into *P*. *tomentosa* and *P*. *nigra* fed after insertion, feeding rate and/or gut retention time could have differed between the two treatments leading to differential retention of environmental microbes in the gut. Additionally, a *Candida* OTU was absent from both poplar treatments, which is often transcriptionally active in the gut and has been previously hypothesized to contribute to the synthesis of branched chain amino acids and metabolism of 5-carbon sugars. Importantly, however, the relative abundance of OTU006 (FSSC) was not impacted by either poplar treatment and no other major changes to the fungal community were noted. Members of FSSC are metabolically versatile and are often capable of metabolizing a variety of aromatic compounds and plant phenolic defensive compounds^29,64–67^ and fungi, in general, appear to be tolerant to salicinoids^68,69^. These factors could lead to the persistence of FSSC and other fungal OTUs in the gut in the two poplar treatments.

Although feeding in *P*. *tomentosa* had more extreme impacts on the richness of the gut bacterial community and on gene expression in the gut compared to feeding in *P*. *nigra*, it is not possible to specifically determine whether the changes to gut gene expression or the microbiota are driving the resistance mechanism in *P*. *tomentosa*. Interactions between xylophagous insects and their microbial symbionts are complex, integrated, and complementary^22,70,71^. As such, disruptions to physiological processes initiated by the insect and/or its  symbionts can negatively impact insect fitness and alter its host plant utilization^10,49,72,73^. Adding further complexity to this system, it is possible that physiological changes within the insect host as a consequence of interacting with its environment elicit reciprocal interactions with symbionts^74^. For example, the altered expression levels of PM and cuticular genes could disrupt molecular interactions between *A*. *glabripennis* and PM-colonizing microbiota. Likewise, having fewer members of the  bacterial community  could reduce the metabolic potential of the community or reduce/eliminate microbial enzyme activity, which could impede the beetle’s ability to access nutrients. Due to the integration of physiological processes, it is often difficult to uncouple the effects of the microbial community and insect physiology on insect fitness without experimental manipulation. However, these results provide microbial targets for future potential manipulation of the *A*. *glabripennis* gut microbial community in gnotobiotic contexts.

Transferring *A*. *glabripennis* larvae from *Acer* spp. into live *P*. *nigra* or *P*. *tomentosa* trees greatly reduced bacterial community richness, altered community structure, and significantly impacted the expression of multi-copy genes with predicted roles in digestion and detoxification. The expression of additional genes related to digestion, detoxification, cuticle/PM, and  development were altered exclusively in insects reared in *P*. *tometosa*, which could be associated with differences in allelochemical profiles between the two poplars. Whether the changes to the gut microbial community or gut gene expression profiles observed in larvae reared in live *P*. *tomentosa* trees could be directly linked to the resistance of this species to *A*. *glabripennis* will require further study. Additionally, the potential roles of defensive compounds in resistance in *P*. *tomentosa* will require further study; however, the avoidance of this species by adult *A*. *glabripennis* is likely a major contributor to the resistance of *P*. *tomentosa* in the field. Overall, the results of this study provides us with an important first step towards identifying factors that enable *A*. *glabripennis* to feed in such a broad host range and the response of the larval stage and its gut community to a tree with documented field-level resistance.

## Materials and Methods

### Larval Rearing in *Populus nigra* and *Populus tomentosa*

Adult *A*. *glabripennis* were reluctant to feed on or oviposit into *P*. *tomentosa*, which is consistent with field observations in China^75^. In order to produce a sufficient number of larvae feeding in *P*. *tomentosa* to complete this study, larvae were manually inserted into both poplar species as reported previously^26,76^. Because the artificial diet used to rear *A*. *glabripennis* in the laboratory contains antimicrobial compounds to protect the diet from spoilage, it can impact the composition of the gut microbial community^26^. To overcome this challenge, field collected insects and live trees were used for these experiments. For this procedure, logs collected from a mixture of infested sugar maples (*Acer saccharum*) and box elders (*Acer negundo*) from a regulated quarantine area in Bethel, OH were transported to a quarantine greenhouse facility at The Pennsylvania State University (University Park, PA). Because the infested trees had been cut into logs as required by the USDA *A*. *glabripennis* eradication program, it was not possible to conclusively determine which logs originated from *A*. *negundo* and *A*. *saccharum;* however, both hosts are highly suitable for *A*. *glabripennis* development^77^.

The *P*. *nigra* and *P*. *tomentosa* trees used in this study were originally purchased as monoclonal saplings from Forest Farms Nursery (Williams, OR). Saplings were planted in 20-gal nursery containers filled with Fafard 52 pine bark medium (Fafard, Agawam, MA) and grown at an outdoor nursery at Penn State University (University Park, PA) until they were 3–4 years old. Approximately four weeks before they were used for experiments, two trees of each poplar species were moved into a USDA-approved quarantine greenhouse (Penn State University, University Park, PA) to allow for acclimation to greenhouse conditions. Trees were approximately 24 cm DBH (diameter at breast height) at the time of the study.

Insects feeding in logs were allowed to acclimate to greenhouse conditions for a period of four weeks prior to removal from the logs. Logs were split and third instars actively feeding in the heartwood were collected. A group of twenty third instars was randomly selected to provide a baseline estimation of the microbial community and gene expression analyses prior to insertion. The remaining sixty third instars were randomized and inserted into two *P*. *nigra* trees and two *P*. *tomentosa* trees^26,76^. The purpose of randomization was to ensure that insects collected from *A*. *negundo* and *A*. *saccharum* were as evenly distributed among the three treatments as possible and only third instars were used in order to standardize developmental stage and minimize ontogenic effects across treatments. After a two week duration, which is the same time period that has been used for previous host-A. *glabripennis* interactions^26^, the larvae were removed from the tree. Larvae were surface sterilized with one rinse of 10% Coverage Plus (Steris Corporation, Mentor, OH) and two rinses of milliQ water. Larval guts were dissected dorsally and the entire gut was collected for further analysis. Only guts from larvae that were alive and actively feeding (indicated by the presence of wood in the gut and frass in tunnels) were selected for further analysis.

### Differential Expression Analysis of Insect-Derived Genes Using RNA-Seq

The impact of feeding in *Acer* spp. (control), *P*. *nigra,* and *P*. *tomentosa* on insect gene expression was assessed using an RNA Seq-based differential expressed analysis. Total RNA was collected from the guts of larvae fed in *P*. *nigra* (n = 3) *P*. *tomentosa*, (n = 3) and control individuals (n = 4) from *Acer* spp. using the Power Microbiome RNA Isolation kit (MoBio, Carlsbad, CA). Sample concentration and integrity were verified with the RNA Nano Assay (Agilent, Santa Clara, WA) and Nano Drop spectrophotometer (Thermo-Fisher, Waltham, MA). Total RNA was poly(A) purified and multiplexed Illumina libraries were constructed using the TruSeq RNA Sample Prep kit (Illumina, San Diego, CA). Samples were pooled and sequenced to a depth of approximately 13 million 101 nt paired end reads per sample on the Illumina HiSeq 2000. Differential expression analysis, GoSeq enrichment analysis, KEGG pathway analysis, and KO assignments were performed as described previously^21^ using the *A*. *glabripennis* gene set v0.5.3 available at ftp://ftp.hgsc.bcm.edu/I5K-pilot/Asian_long-horned_beetle/maker_annotation/version_0.5.3/)^3^. Raw Illumina RNA-Seq reads are deposited in NCBI’s Sequence Read Archive (SRA) under BioProject PRJNA395783 [SRA: SRR5867415–SRR5867424].

### Comparative Microbial Community Analysis

To determine the impact of rearing beetle larvae in the two poplar species on the gut microbial community, DNA was extracted from three randomly selected individual *A*. *glabripennis* larvae recovered from each of the three treatments included in the study using the Power Soil DNA Isolation Kit (MoBio, Carlsbad, CA). DNA concentration and integrity were verified using the Quant-It dsDNA Assay (Life Technologies, Carlsbad, CA) and Nano Drop (Thermo-Scientific, Waltham, MA). DNA collected from each insect was used to construct partial 16S bacterial amplicon libraries ranging from position 27F to 907R, and full ITS amplicon libraries ranging from ITS5 to ITS4 as described previously^23^. ITS and 16S amplifications were performed on the same DNA samples so that the bacterial community and the fungal community from the same individuals were sampled. Amplicon libraries were multiplexed, library titer was verified against a library standard using the 454 qPCR kit (Kapa Systems, Woburn, MA), and samples were sequenced on a 454 Titanium FLX instrument to a depth of approximately 13,000 reads per sample for 16S libraries and approximately 5,000 reads per sample for ITS libraries. A full list of multiplex identifiers used in this study is presented in Supplementary Info [Supplemental Table 2]. Raw reads from each 16S and ITS library are deposited in NCBI’s Sequence Read Archive under the BioProjects PRJNA395947 [SRA: SRR5874813–SRR5874821] and PRJNA395914 [SRA: SRR5871168–SRR5871175] respectively.

### Operational Taxonomic Unit-Based Analysis of 16S and ITS Amplicons

Both 16S and ITS amplicons were assigned to operational taxonomic units (OTUs) using mothur (version 1.32.0)^78^. For analysis of 16S amplicons, high quality reads greater than 700 bp in length were denoised using the shhh.flows command and clustered into operational taxonomic units (OTUs) at 97% similarity using the average neighbor algorithm. Chimeric reads were detected and removed from the dataset using the program UCHIME^79^. Singleton OTUs (i.e., OTUs containing only a single read) were omitted from the analysis. Representative sequences for each OTU were compared to the non-redundant nucleotide database using BLASTN^80^ with an e-value threshold of 0.00001 to detect insect-, fungal-, plant-, chloroplast-, and mitochondrial-derived sequences. OTUs with highest scoring BLAST alignments to non-bacterial taxa or organelles were omitted from the analysis and bacterial reads were classified using the Ribosomal Database Project (RDP) Classifier^81^ with an 80% confidence threshold for taxonomic classifications (Supplementary Data 1).

For analysis of ITS amplicons, high quality reads ranging from 450 bp to 850 bp in length were clustered into OTUs at 97% similarity using a modified procedure. Instead of using multiple pairwise alignments to calculate distance metrics for OTU identification, the program CD-HIT-EST was used to bin sequences with ≥97% similarity into the same OTU using an alignment independent approach. This region is highly prone to indel events^82^ making it difficult to align this region across distantly related taxa and often leading to inflation in the number of predicted OTUs in a sample^83^. Putative OTUs were screened for chimeras using the program UCHIME^79^ and non-fungal sequences were detected and removed from the analysis by a BLASTN comparison^80^ to the non-redundant nucleotide database with an e-value threshold of 0.00001. Taxonomic classification of fungal-derived ITS OTUs was conducted using the UNITE database^84^ using a 90% confidence threshold (Supplementary Data 2).

Prior to performing multivariate bacterial 16S and fungal ITS community comparisons, the libraries were normalized by randomly subsampling (without replacement) the same number of amplicon reads (n = 5,918 for 16S and n = 1,500 for ITS) using the ‘sub.sample’ command in mothur. Richness, diversity, and multivariate analyses were computed using mothur. Additionally, supervised clustering using linear discriminant analysis was conducted using the random forests algorithm (classify.rf command in mothur) to identify OTUs impacted by the two poplar treatments.

### Data availability

Illumina RNA-Seq and 454 16S and ITS amplicon reads are available at NCBI’s Sequence Read Archive under BioProjects PRJNA395783 [SRA: SRR5867415-SRR5867424], PRJNA395947 [SRA: SRR5874813-SRR5874821], and PRJNA395914 [SRA: SRR5871168-SRR5871175], respectively.

## Electronic supplementary material

Supplemental Results

Supplementary Dataset 1

Supplementary Dataset 2
