# Supplementary material for: Host-plant induced changes in microbial community structure and midgut gene expression in an invasive polyphage (Anoplophora glabripennis)
Source: Sci Rep. 2018 Jun 25;8:9620. doi: 10.1038/s41598-018-27476-0 (PMC6018227; doi:10.1038/s41598-018-27476-0)
Supplement: Supplementary file 1 — Supplemental Results [file 41598_2018_27476_MOESM1_ESM.pdf]

Host-plant induced changes in microbial community structure and midgut gene expression in an invasive polyphage (*Anoplophora glabripennis*)

Erin D. Scully[1*]; Scott M. Geib[2]; Charles J. Mason[3]; John E. Carlson[4,5]; Ming Tien[6]; Han-Yi Chen[7‡]; Scott Harding[7,8]; Chung-Jui Tsai[7,8]; and Kelli Hoover[3]

[1]Stored Product Insect and Engineering Research Unit, USDA-ARS Center for Grain and Animal Health Research, Manhattan, KS 66502 USA

[2]Tropical Crop and Commodity Protection Research Unit, USDA-ARS Daniel K. Inouye Pacific Basin Agricultural Research Center, Hilo, HI 96720 USA

[3]Department of Entomology and Center for Chemical Ecology, The Pennsylvania State University, University Park, PA 16802 USA

[4]The Schatz Center for Tree Molecular Genetics, Department of Ecosystem Science and Management, The Pennsylvania State University, University Park, PA 16802 USA

[5]Department of Bioenergy Science and Technology (World Class University), Chonnam National University, Buk-Gu, Gwangju 500-757, Korea

[6]Department of Biochemistry and Molecular Biology, The Pennsylvania State University, University Park, PA 16802 USA

[7]Warnell School of Forestry and Natural Resources, University of Georgia, Athens, GA 30602-2152, USA

[8]Department of Genetics, University of Georgia, Athens, GA 30602-7223, USA

[‡]Present Address: Plants for Human Health Institute, North Carolina State University, Kannapolis, NC 28081, USA

[*]To whom correspondence should be addressed: erin.scully@ars.usda.gov (785) 776-2710

Supplemental Results

Genes Coding for Hemocyanins were Upregulated in the Majority of the Control Larvae (Cluster 5)

Although the expression patterns of cluster five and six each had one outlier in the control (cluster five) or in the *P. nigra* treatment (cluster six), there were several genes in those two modules whose expression levels were altered in the poplar treatment that could have effects on gut physiology. For example, four genes coding for hemocyanins displayed lower expression levels in poplars compared to most of the controls, while genes involved in digestion were also downregulated, including two carboxylesterases and one serine proteinase and/or trypsin inhibitor. Cluster five was enriched in several enriched GO terms, such as ubiquitin-protein transferase activity (GO:004842), carbohydrate metabolic process (GO:0005975), and ion transport (GO:0044765). Other genes assigned to this group included two antimicrobial peptides, one carbonic anhydrase, one HTH DNA binding protein, one OHCU decarboxylase involved in purine catabolism, one choline transporter, one short chain dehydrogenase, transaldolase from the pentose phosphate pathway, and one transferrin involved in iron binding.

Genes Linked to Developmental Processes and Intracellular Signaling were Upregulated in the Majority of the Poplar-Reared Insects (Cluster 6)

Cluster six also contained genes with putative roles in digestion, detoxification, and nutrient acquisition, including two trypsins, two ABC transporters, two MFS transporters, one sulfate transporter, one trehalase, and several genes involved in immunity, including two leucine rich repeats (LRRs). Further, many of proteins encoded by genes assigned to cluster six potentially serve developmental roles including cyclin, glypican, JHBP, patched family protein, pecanex protein, spondin protein, a protein alan shepard homolog, and one RNA binding proten fusilli. In addition, several genes with putative roles in intracellular signaling were also found in

this cluster including a PH domain gene, three protein kinase genes, an adenylate and guanylate cyclase gene, a protein phosphatase gene, a protein tyrosine kinase gene, and two genes encoding the calcium binding proteins calponin and otopetrin. Stress-related genes were also assigned to cluster six including a UDP-glucose:glycoprotein glycosyltransferase, which reglycoslyates un-glycosylated proteins, three apoptotic genes (THAP domain protein, inhibitor of apoptosis, and apoptosis inducing protein), one haloacid dehydrogenase, one HSP70, and one thioredoxin. As in the previous gene clusters, two structural genes were also assigned to this cluster, including genes coding for myosin and ankyrin-rich repeats. Most notably, five transcription factors (TFs) were assigned to cluster six, including one BZIP TF, one zinc-finger TF, two HTH TFs, and one PHD-like TF. Lastly, two genes linked with cholesterol and fatty acid transport were also assigned to this gene cluster, including a gene coding for a CD36 fatty acid transporter and a gene coding for a sterol sensing domain of SREBP cleavage activation.

Supplemental Figures

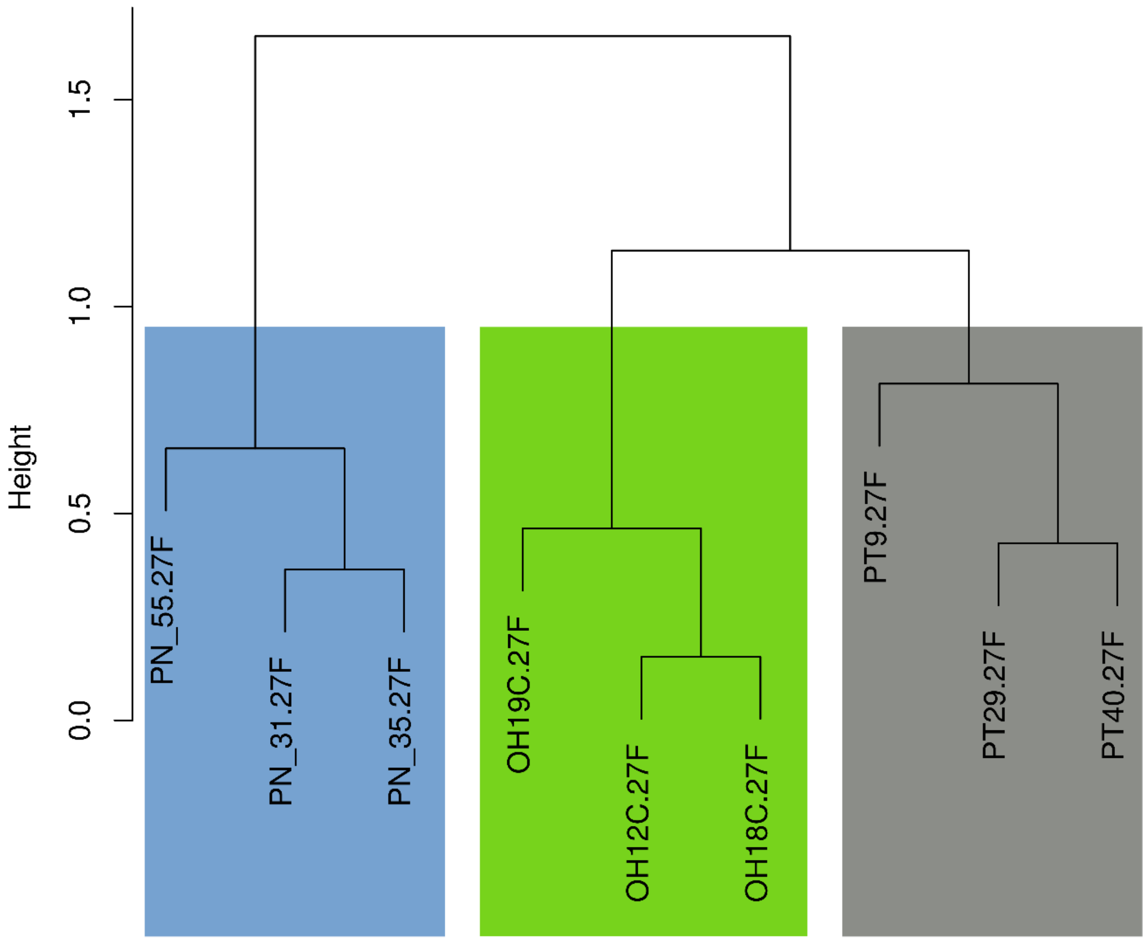

Supplemental Figure 1. Hierarchical Clustering Analysis of 16S Community Compositional Data. In order to validate the clustering results observed in the NMDS plots, hierarchical clustering analysis was performed. Bray-Curtis distances were computed using the 'veg.dist' command from the vegan statistical package with BINARY=TRUE and clustering analysis was performed using Ward's method with the 'hclust' program. As observed in the NMDS plot, the community compositions of the three biological replicates from each treatment. were correlated with one another. Blue=*P. nigra*; Green=Control; Grey=*P. tomentosa*.

Supplemental Tables

Supplemental Table 1. Clusters 5 and 6. Previously, several categories of genes were hypothesized to aid in digestion, detoxification, nutrient acquisition, and host range determination in *A. glabripennis* and expression levels of these genes in the gut were altered as larvae fed on a suitable host compared to a nutrient rich artificial diet high in nutrients and free of plant defensive compounds. Expression levels of several of these genes were altered as larvae fed on poplar. Shown are the cluster assignments and whether or not the expression of these genes was altered in the previous sugar maple versus diet feeding experiment. PBP/GOBP=Pheromone binding protein/general odorant binding protein; ND=no difference; NE=not expressed in sugar maple or artificial diet reared insects.

**Cluster #**	**Gene ID**	**Annotation**	**Expression Altered in Sugar Maple versus Diet Experiment?**	**Direction of Expression Change (SM v Diet)**
5	AGLA000621	Carboxylesterase	Y	Up
5	AGLA002953	Carboxylesterase	N	ND
6	AGLA019046	ABC Transporter	Y	Down
6	AGLA017850	ABC Transporter	N	ND
6	AGLA020448	Insect Cuticle protein	N	NE
6	AGLA010940	Major Facilitator Superfamily	N	NE
6	AGLA003354	Major Facilitator Superfamily	N	NE
6	AGLA019820	Sugar (and other) Transporter	N	NE
6	AGLA007617	Trypsin	Y	Down
6	AGLA004870	Trypsin	N	ND
6	AGLA004754	UDP-Glucuronsyl Transferase	N	ND

Supplemental Table 2. Multiplex identifiers used for 454 16S and ITS amplicon libraries.

**Sample ID**	**Multiplex Identifier**	**Total Read Yield**
	**16S**	
OH12C (Control)	TCGTCGCTCG	13347
OH18C (Control)	ACATACGCGT	12204
OH19C (Control)	ACGCGAGTAT	9764
PN31 (*P. nigra*)	ATATCGCGAG	6502
PN35 (*P. nigra*)	ATATCGCGAG	9928
PN55 (*P. nigra*)	CGTGTCTCTA	9979
PT9 (*P. tomentosa)*	CATAGTAGTG	24169
PT29 (*P. tomentosa)*	CGAGAGATAC	16468
PT40 (*P. tomentosa)*	ATACGACGTA	10231
	**ITS**	
OH12C (Control)	ACGTCTCATC	
OH18C (Control)	ACTCATCTAC	3911
OH19C (Control)	ACTCGCGCAC	9364
PN31 (*P. nigra*)	TGTAGTGTGA	3114
PN55 (*P. nigra*)	TGTCACACGA	6427
PT9 (*P. tomentosa)*	TGTCGTCGCA	1705
PT29 (*P. tomentosa)*	ACACATACGC	9157
PT40 (*P. tomentosa)*	ACAGTCGTGC	1404

Supplementary Data

Supplemental Table 1 (Excel). Normalized read counts and taxonomic assignments for 16S amplicon OTUs.

Supplemental Table 2 (Excel). Normalized read counts and taxonomic assignments for ITS amplicon OTUs.
